# Extended darkness induces internal turnover of glucosinolates in *Arabidopsis thaliana* leaves

**DOI:** 10.1371/journal.pone.0202153

**Published:** 2018-08-09

**Authors:** Saskia Brandt, Sara Fachinger, Takayuki Tohge, Alisdair R. Fernie, Hans-Peter Braun, Tatjana M. Hildebrandt

**Affiliations:** 1 Institut für Pflanzengenetik, Leibniz Universität Hannover, Hannover, Germany; 2 Max-Planck-Institute of Molecular Plant Physiology, Potsdam-Golm, Germany; Indiana University, UNITED STATES

## Abstract

Prolonged darkness leads to carbohydrate starvation, and as a consequence plants degrade proteins and lipids to oxidize amino acids and fatty acids as alternative substrates for mitochondrial ATP production. We investigated, whether the internal breakdown of glucosinolates, a major class of sulfur-containing secondary metabolites, might be an additional component of the carbohydrate starvation response in *Arabidopsis thaliana (A*. *thaliana)*. The glucosinolate content of *A*. *thaliana* leaves was strongly reduced after seven days of darkness. We also detected a significant increase in the activity of myrosinase, the enzyme catalyzing the initial step in glucosinolate breakdown, coinciding with a strong induction of the main leaf myrosinase isoforms TGG1 and TGG2. In addition, nitrilase activity was increased suggesting a turnover via nitriles and carboxylic acids. Internal degradation of glucosinolates might also be involved in diurnal or developmental adaptations of the glucosinolate profile. We observed a diurnal rhythm for myrosinase activity in two-week-old plants. Furthermore, leaf myrosinase activity and protein abundance of TGG2 varied during plant development, whereas leaf protein abundance of TGG1 remained stable indicating regulation at the transcriptional as well as post-translational level.

## Introduction

*Arabidopsis thaliana* contains many secondary metabolites, and glucosinolates (GLSs) are considered to be among the most characteristic [1]. GLSs are synthesized from glucose and amino acids and can be classified into three groups: aliphatic, indole and aromatic GLSs. The ecotype Columbia (Col-0) contains a set of about 30 different GLSs [2]. The main function of GLSs, plant defense against herbivores and pathogens, is mediated by their breakdown products, mainly simple nitriles, epithionitriles and isothiocyanates (ITCs) [3,4]. In particular the hydrolysis product ITC serves as an important defense compound against various plant pests [5,6]. In addition to their function in plant pathogen resistance, GLSs and their breakdown products are also highly relevant for medical research. In cancer cell lines, ITCs were shown to inhibit phase I enzymes, increase the activity of phase II enzymes and to induce apoptotic alterations and cell cycle arrest [7–9]. GLS breakdown is initiated by myrosinase (thioglucoside glucohydrolase, TGG), which hydrolyses the thioglucoside bond to cleave off the glucose group [10,11]. The remaining instable aglucone spontaneously converts to ITC, or in the presence of specifier proteins is metabolized either to a simple nitrile or an epithionitrile [12]. Since GLSs and myrosinases are stored separately in the leaves, this breakdown is thought to occur mainly as a reaction to tissue damage after pathogen attack [13,14]. However, several studies also indicate that there might be an internal turnover of GLSs in intact plant tissues [12,15–20]. First, the tissue GLS content as well as composition constantly changes throughout the life span of *A*. *thaliana*. Seeds have a particularly high GLS content with a characteristic composition. They contain mainly GLSs without secondary modifications while the modified versions are more abundant in vegetative tissues [2,21,22]. Conversion from the seed to the leaf GLS set requires degradation of the seed GLSs during the early seedling developmental process, which has been confirmed using a radiolabeled GLS [21]. GLS contents then increase in leaves until the bolting stage and decrease again during senescence, which most likely also involves internal degradation [2,21]. Secondly, the total GLS content of *A*. *thaliana* leaves shows diurnal fluctuations, and the decrease detected during the night might be due to internal turnover [23, 24]. Thirdly, a decrease in GLS content observed during sulfur depletion indicates that GLSs might serve as sulfur storage compounds [25–28].

In the roots, atypical myrosinases such as PYK10 (At3g09260) and GLSs are stored in two independent compartments of the same cell, the ER bodies and vacuoles, respectively [29]. Thus, GLS turnover could take place without tissue disruption following translocation of myrosinases into vacuoles, co-secretion of myrosinases and GLSs out of the cell or single cell collapse. In leaves, GLSs are stored primarily in specialized S-cells localized in the midvein close to the phloem. The main leaf myrosinase isoforms TGG1 and TGG2 are expressed in scattered myrosin cells as well as in phloem-associated cells, and TGG1 in addition is highly abundant in stomatal guard cells (reviewed by [12]). Subcellular localization is not entirely clear yet. Binding of MVP1 (Modified vacuolar phenotype 1; At1g54030) to TGG2 was suggested to translocate TGG2 relative to GLSs and thus enable turnover in intact leaves [12]. Inducible ER bodies containing several atypical myrosinases have also been detected in *A*. *thaliana* leaves [30–32]. However, their physiological role in glucosinolate metabolism has not been established.

Tissue disruption leads to specific profiles of GLS breakdown products in the different *A*. *thaliana* ecotypes and also in individual plant organs. In addition, GLS hydrolysis products change dynamically during development as well as in response to specific environmental conditions and pathogen attack [33–35]. Col-0 rosettes produce primarily ITCs, but nevertheless express functional specifier proteins and are capable of producing at least low levels of simple nitriles [34–37]. For efficient nutrient remobilization, the GLS breakdown products should be non-toxic and easy to integrate into primary plant metabolism. Thus, the degradation pathway via simple nitriles seems to be the most likely option for internal GLS turnover. Nitriles can be further catabolized via nitrilases into carboxylic acids. Via this pathway glucose, sulfate, elemental sulfur, and nitrogen in form of ammonium are released, and all of these nutrients can be reused in primary metabolism or as energy supply [17,38,39].

The aim of the present study was to analyze internal turnover of GLSs in *A*. *thaliana* leaves under clearly defined conditions. Total myrosinase activity in combination with the protein abundance of the major myrosinase isoforms expressed in leaves (TGG1 and TGG2) were used as a marker for the induction of GLS breakdown. We also measured nitrilase activity in order to estimate the potential flux through this branch of the GLS degradation pathway. A developmental timeline and a diurnal setup were included since changes in the GLS profile have been reported under these conditions. In addition, we used extended darkness as a tool to induce carbohydrate starvation. In the absence of photosynthesis, plants remobilize nutrients such that they can use amino acids and fatty acids as alternative substrates for ATP production [40,41]. Our results indicate that GLS turnover might also be induced during extended darkness to provide nitrogen and sulfur in addition to glucose and carboxylic acids as substrates for ATP production most likely via the nitrile pathway.

## Material & methods

### Plant material and growth conditions

All plants used for this study were *Arabidopsis thaliana* ecotype Columbia (Col-0). The plants were grown under long day (LD; 16 h light/8 h dark) and short day (SD; 8 h light/16 h dark) conditions at 22 °C with a light intensity of 85 μmol s^−1^ m^−2^ light and 65% humidity. Rosette leaves were used for all experiments. The plant material was frozen in liquid nitrogen immediately after harvest, ground to a fine powder, and stored at -80°C until use.

The diurnal setup was performed with two-week-old LD plants (pooled to four to five replicates) and the four harvest points were: (i) at the beginning of the light period, (ii) after 8 h of light, (iii) at the end of the light period and (iv) after 4 h of darkness. Plants at different developmental stages were harvested at the beginning of the light period. For the measurements, four to five plants from each developmental stage were taken and pools collected of two- and three-week-old LD and three- and four-week-old SD plants, respectively.

Extended darkness experiments were performed with 42 d old SD plants (four to five replicates). The plants were harvested after 3 d (45 d old plants) and 7 d (49 d old plants) of darkness. Metabolite data were obtained from plants grown for 42 d under SD conditions followed by one week of darkness.

### GLS profiling

Metabolites were extracted by adding 80% methanol to the frozen leaf powder kept at liquid nitrogen temperature to reach 0.2 mg FW μl^-1^ and homogenizing with a mixer mill for 2 min [42]. Profiling of GLSs by LC-MS was performed using a Surveyor HPLC system source coupled to a linear ion trap (IT) ESI-MS system FINNIGAN-LTQ (Thermo Finnigan, USA) mass detector according to the previously published protocol [42]. The HPLC system was equipped with a Luna C18(2) reversed phase column (150 x 2.0 mm i.d. 3.0 μm particle size, Phenomenex). The mobile phases were solvent A: 0.1% formic acid in water and, solvent B: 0.1% formic acid in acetonitrile. The elution flow rate of the mobile phase was 200 μl/min, and 2μl sample were loaded per injection. The LTQ Linear ion trap MS with a heated electro spray source was used with full scan mode of negative and positive ion detections, covering a mass range from *m/z* 200–1500. Chromatographic data were processed using Xcalibur 2.2 software (Thermo Fisher Scientific). The processed data matrix was normalized using an internal standard (Isovitexin; CAS 29702-25-8) in extraction buffer (5 μg ml^-1^). For data analysis, relative peak areas representing mass spectral ion currents were normalized to sample fresh weight fresh weight (FW) and peak area of the internal standard. Metabolites were identified and annotated based on previously published data [43], as well as the properties of purified compounds obtained from *A*. *thaliana* extracts [44].

### Myrosinase activity

The method from Palmieri et al., 1982 [45] was used to determine the myrosinase activity. For extraction 600 μl of ice-cold potassium phosphate buffer (25 mM, pH 7.0) was added to 40 mg of frozen and ground rosette leaves, vortexed and kept on ice for 10 min. After centrifugation (10 min; 10000 *g*), 450 μl of the supernatant was loaded onto Microcon^®^ Centrifugal Filter Devices with a cutoff of 30 kDa. After two filtering steps and filling up the supernatant in the filter devices to 500μl in between with potassium phosphate buffer, 75 μl of the extract was used for the activity measurement. A total volume of 300 μl was composed of extraction buffer, 0.13 mM ascorbic acid, 0.5 mM sinigrin and 75 μl of the extract. The absorbance of sinigrin was measured photometrically in a UV-permeable 96 well plate at 227 nm for 15 min. Each 9 sec a data point was collected and rates were taken in the linear phase. For each biological replicate (measured in duplicate), a control with extraction buffer, 0.13 mM ascorbic acid and 75 μl of the extract was measured and the obtained rates were substracted from the samples measured with 0.5 mM sinigrin. The activity was calculated using the extinction coefficient of Ɛ_227_ = 7273 M^-^1 cm^-1^ for sinigrin [22].

### Nitrilase activity

For the measurement of nitrilase activity, the production of ammonia was measured by the Berthelot reaction [46]. Approximately 60 mg of frozen and ground plant material was extracted with 500 μl of 100 mM sodium phosphate buffer (ice cold) and kept on ice for 10 min. After centrifugation, the supernatant (100 μl) was taken directly or heated for 10 min at 100 °C (control). The nitrilase reaction was started by addition of the substrate 6-heptenenitrile (2.5 mM) and samples were incubated at 30 °C for 45 min. For detection of the produced NH_3_, 330 mM sodium phenolate trihydrate, 20 mM sodiumhypochloride and 0.01% disodium pentacyanonitrosyl ferrate (III) dihydrate (sodiumprussid) were added and heating at 99 °C for 2 min was performed. Samples were diluted with 600 μl H_2_O and analysed in a 96 well plate at 640 nm. Each sample was measured in triplet. The activity was calculated using a standard curve with NH_4_Cl. The protein content of the samples was determined using the Pierce Coomassie (Bradford) Protein Assay (Thermo Fisher Scientific).

### Western blots

A denaturing SDS-PAGE (Biorad; Mini-PROTEAN^®^ TGX Stain-Free^™^ Precast Gel) was performed (45 mA; 40 min) with 6 μg of leaf extract. As a marker, the Amersham ECL High-Range Rainbow marker (GE Healthcare) was used. The ensuing blotting to a nitrocellulose membrane was performed for 1.5 h at 440 mA and the proteins were detected by anti-TGG1 and anti-TGG2 antibodies [14]. Coomassie staining and a polyclonal anti-actin antibody (Agrisera; AS13 2640) were used as a loading control. Detection was performed by the usage of a secondary antibody goat-anti-rabbit conjugated with horseradish-peroxidase (Agrisera) and the Amersham ECL Western Blotting Detection Reagent (GE Healthcare). All experiments were performed in duplicate or triplicate.

### Statistical analyses

Data are given as means ± standard deviation. Significant differences between means were evaluated by either Student’s T-tests or Student Newman Keuls tests at the p < 0.05 level using a statistical software package (XLStat Base, Addinsoft Paris, France).

## Results

### Myrosinase activity during plant development

**Fig 1:**
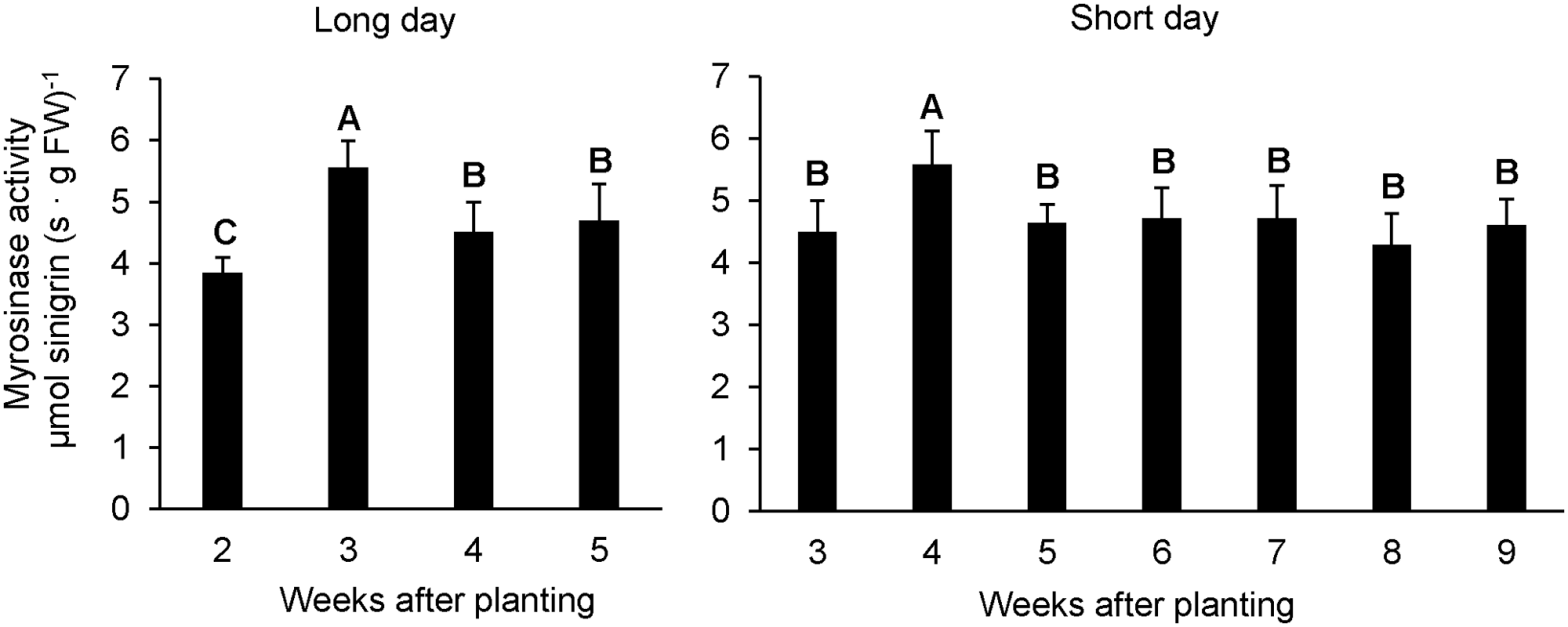
Myrosinase activity in rosette leaves during development of *A*. *thaliana*. Complete rosettes were harvested at the beginning of the light period after 2–5 weeks of growth under long-day conditions (16/8h light/dark), and 3–9 weeks of growth under short-day conditions (8/16 h light/dark). Error bars indicate the standard deviation of four to five biological replicates or pools. Significantly different means are indicated by different letters (Student Newman Keuls; P < 0.05).

**Fig 2:**
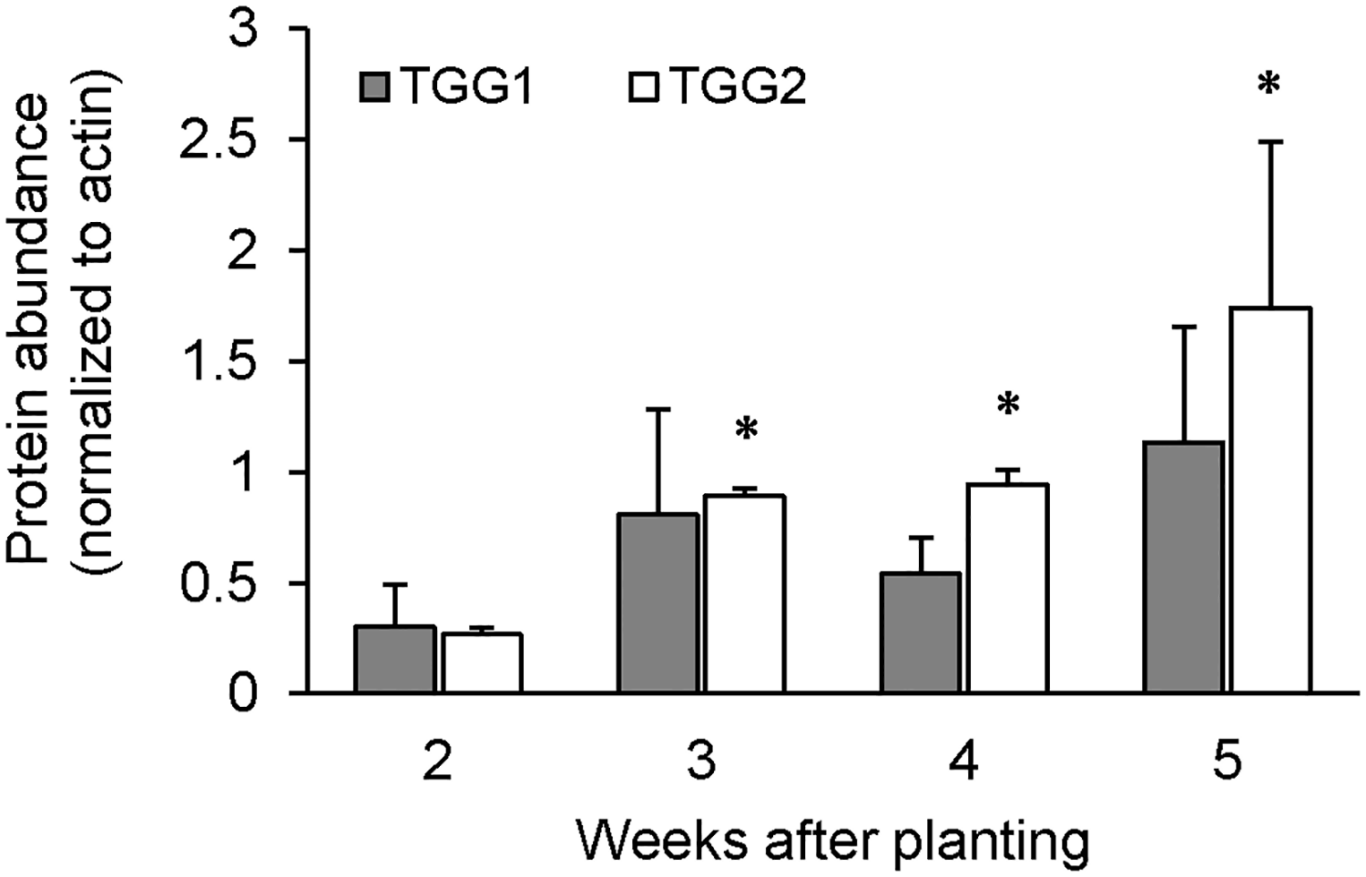
Protein abundance of TGG1 and TGG2 during development of *A*. *thaliana* plants grown under long-day conditions. Rosette leaves were harvested at the beginning of the light period and protein abundance was quantified after western-blotting and immunodetection using ImageJ. Asterics indicate significant differences (Student’s T-test; P < 0.01) compared to the protein abundance in two-week-old plants. Error bars indicate the standard deviation of two to three independent experiments with pools (2- and 3-week-old plants) and single plants (4- and 5-week-old plants).

In order to identify a possible turnover of GLSs in rosette leaves, we tested the myrosinase activity in two- to five-week-old plants grown under long-day (LD) conditions and additionally in plants grown under short-day (SD) conditions for three to nine weeks (Fig 1 and S1 Fig). Plants grown under LD conditions showed an increased myrosinase activity at the age of three compared to two weeks and this activity subsequently decreased during further development (Fig 1). We tested abundances of the myrosinase isoforms TGG1 and TGG2 to estimate if the higher activity was due to a higher protein content in the leaves. The abundance of TGG1 remained stable in two- to five-week-old plants, while TGG2 was increased in three- to five-week-old plants (Fig 2).

Col-0 plants produced more leaf biomass under SD conditions compared to LD conditions and started to bolt much later, such that nine-week-old SD plants had not yet entered the bolting stage. The leaf myrosinase activity was constant in three- to nine-week-old plants except for a peak at the age of four weeks (Fig 1). Taking together the results from the myrosinase activity measurement and the phenotype, five- to nine-week-old plants grown under SD conditions provide a stable background to investigate the effect of stress treatments on myrosinase activity in *A*. *thaliana*.

### Myrosinase activity during the diurnal cycle

**Fig 3:**
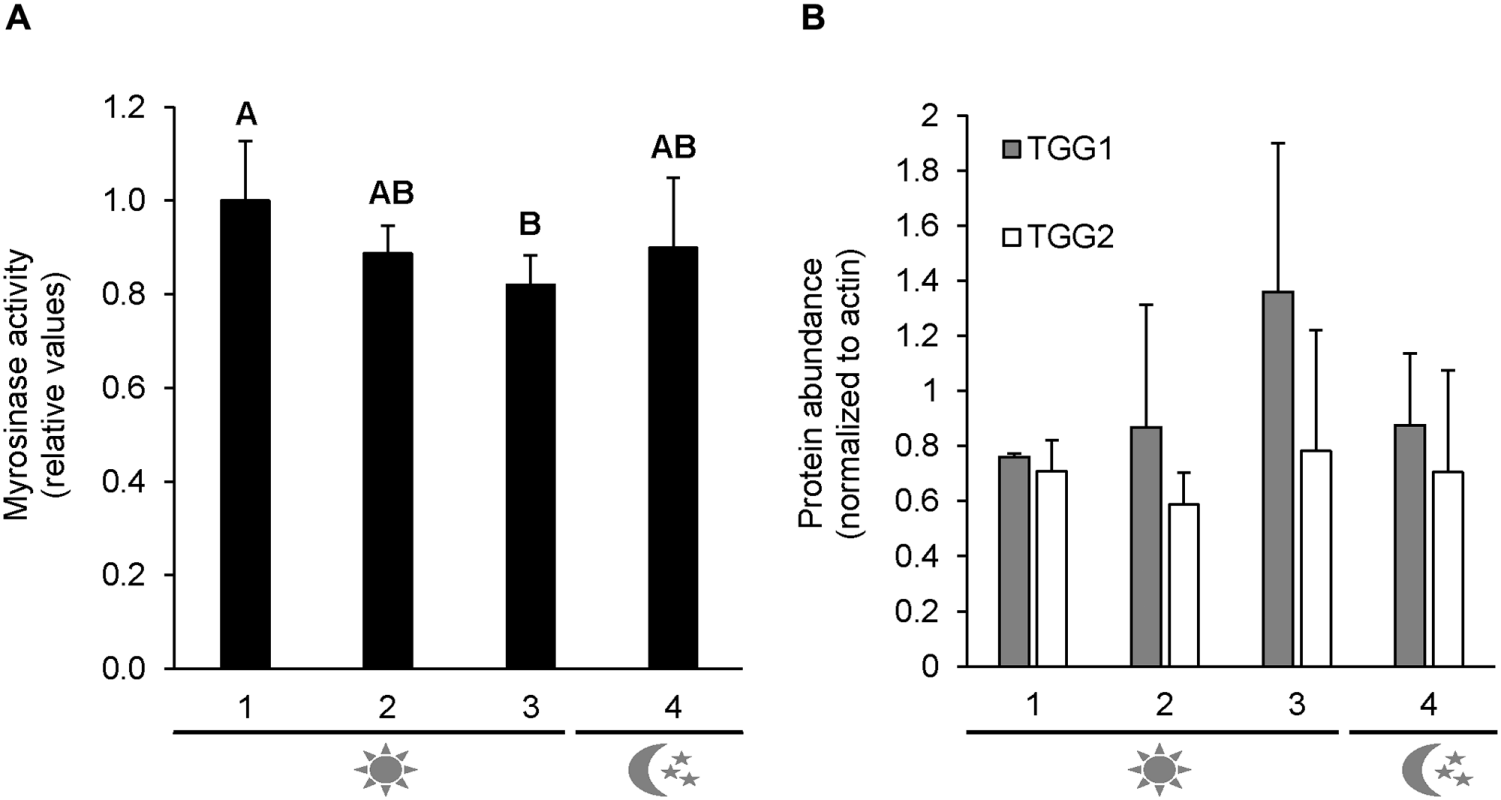
Diurnal myrosinase activity and protein abundance in two-week-old *A*. *thaliana* plants grown under long-day conditions. The plants were harvested at four different time points: **1** Beginning of the light period; **2** Middle of the day; **3** End of the light period; **4** Middle of the night. The myrosinase activity (**A**) was measured photometrically and normalized to the maximal activity. Error bars show the standard deviation of four to five biological replicates (pools). For protein abundance of TGG1 and TGG2 (**B**), immunoblotting and normalization with actin was performed. Error bars represent the standard deviation of three independent experiments with one pool of plants. Significantly different means are indicated by different letters (Student Newman Keuls; P < 0.05).

Huseby et al. (2013) [23] demonstrated a diurnal synthesis of GLSs in leaves of two-week-old *A*. *thaliana* plants grown under LD conditions. We tested, whether GLS catabolism also followed a diurnal rhythm under these growth conditions and detected a significant decrease in myrosinase activity during the light period and an increase in the dark (Fig 3a). Western blot analyses showed no significant variation in the protein abundance for the myrosinase isoforms TGG1 and TGG2 during the diurnal cycle (Fig 3b), indicating post-translational activation of myrosinases in the light or deactivation in the dark.

### Glucosinolate turnover under extended darkness conditions

**Fig 4:**
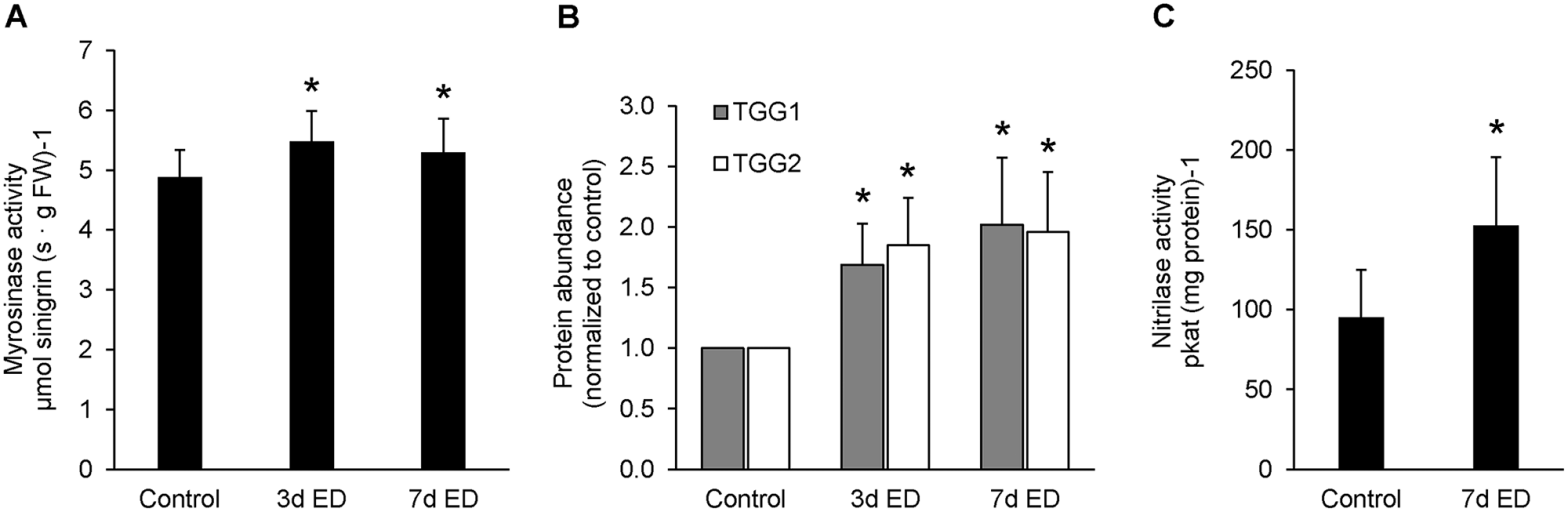
Myrosinase activity, myrosinase protein abundance and nitrilase activity in rosette leaves after extended darkness (ED). The plants were transferred to complete darkness for 3 d (**3d ED**) or 7 d (**7d ED**) after six weeks of growth under short day conditions. Myrosinase (**A**) as well as nitrilase (**C**) activity was measured photometrically (error bars show the standard deviation of five to 19 biological replicates). For protein abundance of TGG1 and TGG2 (**B**), immunoblotting and normalization to the control was performed (error bars represent the standard deviation of three biological replicates from three independently performed Western blots). Asterics indicate significant differences (Student’s T-test; P < 0.05) compared to the control.

Carbohydrate starvation was induced by extended darkness (ED) treatment of *A*. *thaliana* plants for three days (3 d) or seven days (7 d). Plants grown for six weeks under short-day conditions were selected for this experiment since myrosinase activity is stable (Fig 1) and plants have produced sufficient leaf material for analysis (S2 Fig). Myrosinase activity and the protein abundance of both myrosinase isoforms were enhanced after 3 d and 7 d of ED (Fig 4a). TGG1 and TGG2 were increased 1.7-fold and 1.8-fold, respectively, after 3 d and 2-fold after 7 d of ED (Fig 4b and S3 Fig).

**Fig 5:**
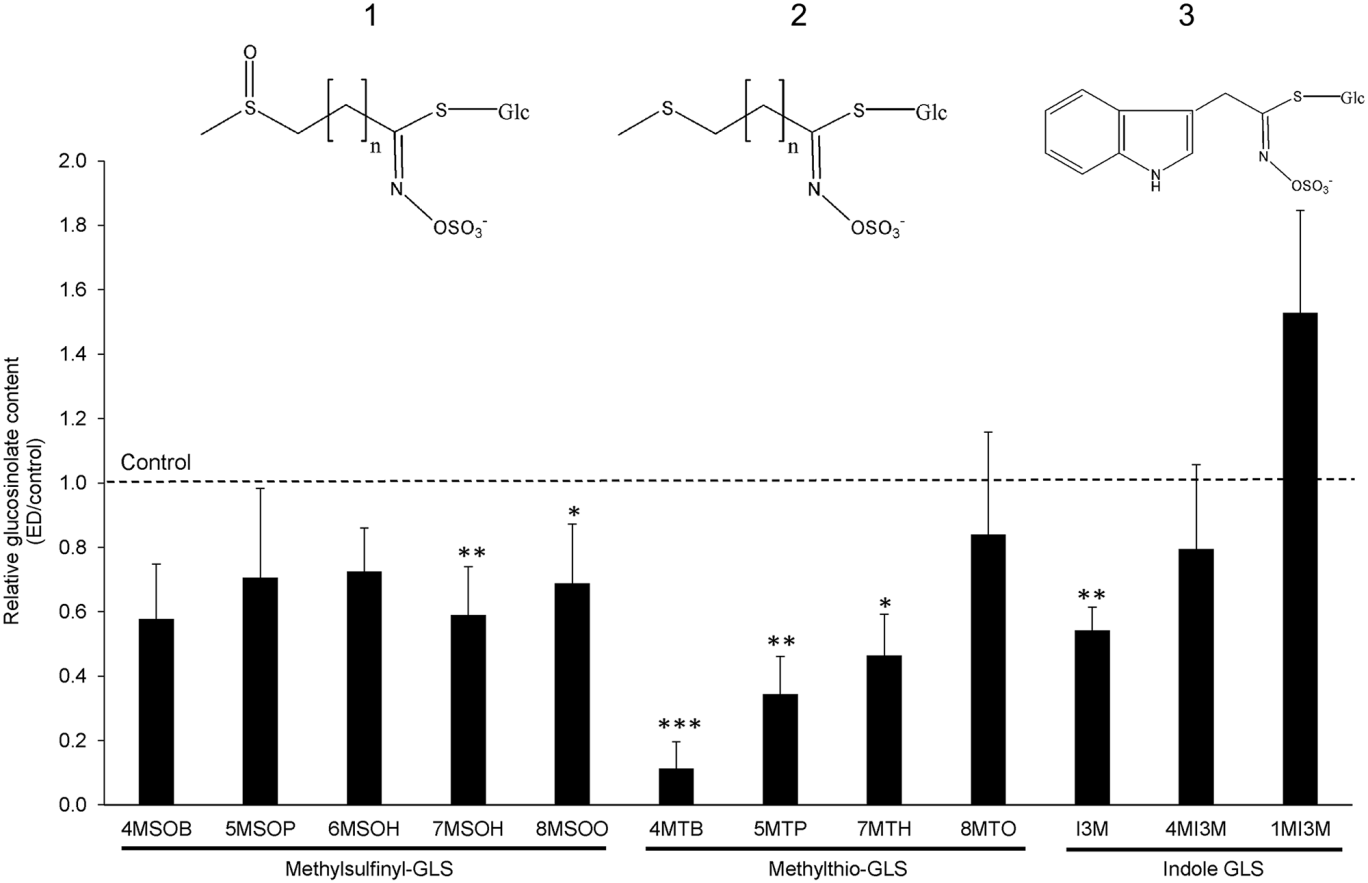
Glucosinolate content in rosette leaves after extended darkness (ED). Plants were grown for six weeks under short day condition and transferred to darkness for 7 d. Glucosinolate content of rosette leaves was measured via LC-MS. Values are means of three to four biological replicates (error bars represent the standard deviation) and Student’s T-test shows *P < 0.05; ** P < 0.01; ***P < 0.001. **1**: general structure of methylsulfinyl-GLS; **2**: general structure of methylthio-GLS; **3**: structure of I3M. **4MSOB** (4-Methylsulfinylbutyl-GLS); **5MSOP** (5-Methylsulfinylpentyl-GLS); **6MSOH** (6-Methylsulfinylhexyl-GLS); **7MSOH** (7-Methylsulfinylheptyl); **8MSOO** (8-Methylsulfinyloctyl); **4MTB** (4-Methylthiobutyl-GLS); **5MTP** (5-Methylthiopentyl-GLS); **7MTH** (7-Methylthioheptyl-GLS); **8MTO** (8-Methylthiooctyl-GLS); **I3M** (Indol-3-ylmethyl-GLS); **4MI3M** (4-Methoxy-indol-3-ylmethyl-GLS); **1MI3M** (N-Methoxy-indol-3-ylmethyl-GLS).

The enhanced leaf myrosinase activity and the higher abundance of TGG1 and TGG2 indicated a turnover of GLSs. To provide further evidence, the contents of GLSs in leaves of *A*. *thaliana* were measured by LC-MS. Results showed up to 89% reduced contents mainly of aliphatic GLSs in rosette leaves after one week of darkness (Fig 5). The strongest decrease (89%) was observed for 4-methylthiobutyl-GLS (4MTB), followed by 5-methylthiopentyl-GLS (5MTP, 66% decrease) and 7-methylthioheptyl-GLS (7MTH, 54% decrease). This shows that especially methylthio-GLS contents were decreased in leaves. In addition, the content of 7-methylsulfinylheptyl-GLS (7MSOH) was reduced by 41% and 8-methylsulfinyloctyl-GLS (8MSOO) was reduced by 31%. Indol-3-ylmethyl-GLS (I3M) was the only indole GLS which showed a decreased content, being depleted by 46%. We used a published GLS profile of Col-0 rosette leaves in the vegetative state [2] to estimate the effect of ED on the absolute GLS contents (S4 Fig). Assuming a similar profile in our plants the total GLS content of the rosette leaves would have decreased from 16.3 μmol g DW^-1^ to 9.6 μmol g DW^-1^ after seven days in the dark.

The decrease of specific GLSs in addition to an increase in protein abundance of TGG1 and TGG2 and an enhanced myrosinase activity in leaves indicates that turnover of GLSs might take place under ED conditions. We also measured nitrilase activity in order to estimate, whether breakdown into carboxylic acids via simple nitriles could be relevant for this process. Nitrilase activity was indeed significantly induced in leaves kept for one week under ED conditions compared to the control (Fig 4c).

## Discussion

### Myrosinase activity but not protein abundance peaks in young *A*. *thaliana* plants

Our results indicate that total leaf myrosinase activity reaches a maximum when plants are approximately at the ten-leaf rosette stage (stage 1.10, [47]), (Fig 1 and S1 Fig). Since growth and development of *A*. *thaliana* plants is delayed under SD compared to LD conditions, this developmental stage is reached after four and three weeks, respectively. A previous study addressing developmental changes in myrosinase activity under LD conditions produced similar results [22]. Remarkably, the protein abundance of the myrosinase isoform TGG2 was significantly enhanced from three to five weeks compared to two-week-old plants possibly indicating a growing importance of this isoform in mature plants. Petersen and colleagues (2002) [21] also showed a high increase in myrosinase content from young to mature bolting Col-0 plants using a 3D7 antibody against *Brassica napus* myrosinase. This antibody turned out to be specific for TGG2 [14] and therefore the results of Petersen et al. (2002) [21] were comparable with the findings we report here. Furthermore, Barth and Jander (2006) [22] demonstrated an increased myrosinase activity in mature plants in the *tgg1* mutant. Thus, not only the protein abundance, but also the activity of TGG2 was enhanced in mature Col-0 plants, indicating possible regulation at the transcript level. Using specific antibodies against TGG1 and TGG2, respectively, we could additionally determine the protein abundance of TGG1, which was not altered during development. Total myrosinase activity decreased in four- and five-week-old plants, while the protein abundances of TGG1 and TGG2 remained stable. Therefore, myrosinase activity of TGG1 and TGG2 would additionally appear to be regulated post-translationally. Another explanation could be the additional activity of atypical myrosinases in leaves of three- to five-week-old plants.

### Diurnal regulation of leaf myrosinase activity

The synthesis of GLSs requires amino acids, glucose, activated sulfur and ATP. Sulfate assimilation as well as GLS synthesis show a diurnal regulation pattern in two week old Col-0 plants grown under long-day conditions with maximal rates during the day when precursors and reducing power are available from photosynthesis [23]. Our dataset indicates that GLS turnover might be regulated reciprocally leading to increased degradation rates during the night. At least under certain growth or developmental conditions GLS breakdown could thus contribute to the reallocation of energy or nutrients during the dark period, as has been shown for amino acid metabolism [48,49]. Indeed, the GLS content increased during the day and decreased again during the night in *A*. *thaliana* plants as well as in postharvest cabbage (*Brassica oleracea*) supporting this hypothesis [23, 24]. The changes in GLS content followed a biphasic pattern even after the plants had been shifted to constant light conditions indicating circadian regulation [24]. Indeed, storage under light/dark cycles has been shown to improve the content of potentially health-promoting GLSs in leafy vegetables [50]. Given that neither TGG1 nor TGG2 showed a diurnally altered protein abundance, we postulate that the observed changes in myrosinase activity are mediate by an as yet unknown post-transcriptional regulation mechanism.

### Extended darkness induces internal turnover of glucosinolates in *A*. *thaliana* leaves

Extended darkness leads to carbohydrate starvation, and as a consequence plants degrade proteins and lipids in order to oxidize amino acids and fatty acids as alternative substrates for mitochondrial ATP production [40]. Our results suggest that GLS breakdown in the leaves might be an additional component of the carbohydrate starvation response in *A*. *thaliana*. Total leaf myrosinase activity was moderately increased after 3 and 7 days of darkness. This was accompanied by a two-fold increase in TGG1 and TGG2 protein content. The finding that TGG1 and TGG2 are not only excepted from degradation but even synthesized during the extended darkness period shows that leaf myrosinases have a crucial function for survival during carbohydrate starvation. The most obvious potential benefit from GLS turnover, the supply of oxidizable substrates for ATP production, could best be achieved via the nitrilase-dependent degradation pathway. GLS breakdown by this pathway releases glucose and either carboxylic acids or the corresponding amids without producing toxic by-products such as ITCs [51]. However, the specific set of products resulting from the degradation of individual GLSs still needs to be established. Col-0 plants express five different isoforms of nitrile-specifier protein and four nitrilases [12]. Products of GLS catabolism are the preferred substrates of the nitrilase isoforms NIT1, 2 and 3, whereas NIT4 is a β-cyanoalanine hydrolase involved in cyanide detoxification [52]. Our data reveal a significant increase in total leaf nitrilase activity during extended darkness and, taken together with the elevated myrosinase activity, indicate a potential role of this pathway in internal GLS turnover. Wounding of Col-0 rosette leaves leads to rapid GLS breakdown resulting mainly in the production of ITCs, which are beneficial during pathogen attack since their high toxicity makes them efficient defense compounds [33, 34, 36]. Under these conditions, high amounts of specifier proteins would be required to redirect GLS breakdown quantitatively towards nitrile production. In contrast, endogenous catabolism in intact cells is a much slower process so that relatively low copy numbers of modifying proteins are sufficient for shifting the balance towards nitriles. NSP dependent GLS degradation to simple nitriles has been demonstrated in Col-0 rosettes and the observed increase with plant age is in line with a postulated function of this pathway in internal GLS turnover [35, 37].

Several scenarios are possible that would enable internal GLS degradation within intact leaves. First, there might be distinct pools of myrosinase, e.g. a large one in the M-cells for fast GLS breakdown after tissue disruption (the “mustard bomb”, [53]) and a second smaller, potentially inducible pool in the S-cells which catalyzes a more gradual GLS turnover in intact tissue [54]. Secondly, translocation of myrosinase by specific mediator proteins such as MVP1 has already been suggested to affect the subcellular localization of TGG2 [12]. Thirdly, internal turnover might be a specific function of one of the myrosinase isoforms. However, the finding that both major leaf myrosinase isoforms TGG1 and TGG2 are strongly up-regulated during carbohydrate starvation renders this scenario rather unlikely.

A study addressing the role of light in the regulation of GLS biosynthesis in *A*. *thaliana* had previously detected a decrease in total GLS content by 25% after 44 hours of darkness [23]. Since the plants were still in an early developmental stage, which is characterized by net synthesis and accumulation of GLSs, this effect might have been caused by a decrease in GLS synthesis rates in the dark treated plants compared to the light grown controls, increased GLS turnover, or a combination of both factors. In our approach, plants grown under short-day conditions for 6 weeks were used. These are in a vegetative growth stage with a stable myrosinase abundance and activity and therefore most likely no major changes in the GLS profile occur at this time point. Thus, the decrease in relative leaf GLS levels after 7 days of extended darkness can likely be attributed predominantly to internal degradation. This finding is well in line with the induction of myrosinase abundance and activity in the leaves. However, GLSs are also actively transported in the phloem and in the xylem [55, 56]. Thus, it cannot be excluded that repartitioning into the roots also contributes to the observed changes in leaf GLS profiles.

Interestingly, we detected large differences between the relative turnover rates of individual GLSs (Fig 5). Short-chain methylthio-GLSs representing the unmodified GLS core structure (4MTB, 5MTP, 7MTH) were reduced most markedly, whilst a less pronounced but still significant decrease was detected for the long-chain methylsulfinyl-GLSs (7MSOH and 8MSOO) and indolic I3M. Unbiased degradation of a mixture of GLSs would be anticipated to lead to equal relative decreases in all glucosinolates. The disappearance of methylthio-GLSs might at least partially be caused by S-oxygenation to the corresponding methylsulfinyl-GLSs by the action of flavin-containing monooxygenases even though we did not detect an accumulation of the oxidized GLSs during extended darkness [57–59]. Strikingly, the set of GLSs showing the largest decrease rates corresponds exactly to the characteristic seed GLSs profile, where 4MTB is most abundant [2,21,22]. Thus, another possible explanation for our results would be that the enzyme set catalyzing internal GLS turnover might be optimized for converting the seed GLS profile to the totally different composition present in the leaves during early seedling growth. This process would require a certain substrate specificity of myrosinases. TGG1 and TGG2 expressed in *E*. *coli* or *Pichia pastoris* have been shown to metabolize different GLSs with different rates [60]. However, preferences seem to be similar in both isoforms and seed specific GLSs have not been tested [60]. TGG1 and TGG2 also do not show specificity for particular GLSs in crushed tissue [22], so additional regulatory proteins might be involved in internal GLS turnover *in vivo*.

It has to be borne in mind that, while GLS breakdown in leaf homogenate was severely decreased in *tgg1*/*tgg2* double knockout mutants, the developmental decrease in GLS content during germination and senescence was unaffected [22]. Thus, there must be an alternative route to catalyze the much slower internal GLS degradation process, which could also be relevant during extended darkness. The fate of the indole GLS I3M during internal degradation might be another interesting aspect to address in future studies given that in the presence of nitrile specifier proteins myrosinase can catabolize I3M to the nitrile indole-3-acetonitrile, which is further degraded by nitrilases into indole-3-acetic acid (IAA) the plant hormone auxin [61–63].

## Conclusion

In this study, we demonstrated that leaf myrosinase activity changes during plant development as well as diurnally most likely due to a combination of transcriptional and post-translational regulation mechanisms. Extended darkness strongly induces both major leaf myrosinase isoforms TGG1 and TGG1 as well as total nitrilase activity and leads to internal GLS degradation. The knowledge concerning GLS turnover and its regulation is of special interest regarding the chemo-preventive properties of GLS breakdown products (for review see [8,64]). We identified extended darkness as a means to induce turnover of specific GLSs. Further elucidation of the mechanism conveying myrosinase substrate specificity will be a useful step into the direction of producing plants that contain a particularly beneficial GLS profile for human health.

## Supporting information

S1 Fig Phenotype of developmental stages under long day and short day conditions. The plants were grown at 22 °C, 85 μmol s^−1^ m^−2^ light and 65% humidity for 16 h light/8 h dark (long day) and 8 h light/16 h dark (short day). Numbers indicate the age of the plants in weeks.
(TIF)

S2 Fig Plants grown under extended darkness (ED) conditions for 3 d and 7 d. Phenotype of six week old plants grown under short-day conditions (22 °C, 85 μmol s^−1^ m^−2^ light, 65% humidity) and transferred to darkness (22 °C, 65% humidity) for 3 d and 7 d.
(TIF)

S3 Fig Coomassie-stained SDS-gels and Western blots of TGG1 and TGG2. Leaves of six-week-old plants, grown under short day conditions, were harvested after 0 d (**Control**), 3 d (**3 d ED**) and 7 d (**7 d ED**) of extended darkness, directly frozen and ground to powder. 6 μg of denatured leaf extract was load on a SDS-PAGE and further either stained with Coomassie brilliant blue (**Coomassie 1/2**) or transferred to a nitrocellulose membrane (**Western Blot 1/2**). Specific antibodies against the myrosinase isoforms TGG1 and TGG2 were used. **M**: Amersham ECL High-Range Rainbow marker (GE Healthcare). **A-C**: three biological replicates. The samples for Coomassie-stain and the Western Blots were prepared on one gel for TGG1 and TGG2, respectively.
(TIF)

S4 Fig Estimation of absolute glucosinolate contents in rosette leaves after extended darkness (ED). The GLS profile of rosette leaves in the vegetative state published by Brown et al. 2003 [2] (blue bars) was used to calculate the theoretical content of each GLC analyzed in this study after 7d of extended darkness (red bars). Relative changes in GLS levels including standard deviations and statistics are shown in Fig 5. 4MSOB (4-Methylsulfinylbutyl-GLS); 5MSOP (5-Methylsulfinylpentyl-GLS); 6MSOH (6-Methylsulfinylhexyl-GLS); 7MSOH (7-Methylsulfinylheptyl); 8MSOO (8-Methylsulfinyloctyl); 4MTB (4-Methylthiobutyl-GLS); 5MTP (5-Methylthiopentyl-GLS); 7MTH (7-Methylthioheptyl-GLS); 8MTO (8-Methylthiooctyl-GLS); I3M (Indol-3-ylmethyl-GLS); 4MI3M (4-Methoxy-indol-3-ylmethyl-GLS); 1MI3M (N-Methoxy-indol-3-ylmethyl-GLS).
(TIF)

## Abbreviations

1MI3M: N-Methoxy-indol-3-ylmethyl-GLS
4MI3M: 4-Methoxy-indol-3-ylmethyl-GLS
4MSOB: 4-Methylsulfinylbutyl-GLS
4MTB: 4-Methylthiobutyl-GLS
5MSOP: 5-Methylsulfinylpentyl-GLS
5MTP: 5-Methylthiopentyl-GLS
6MSOH: 6-Methylsulfinylhexyl-GLS
7MSOH: 7-Methylsulfinylheptyl
7MTH: 7-Methylthioheptyl-GLS
8MSOO: 8-Methylsulfinyloctyl
8MTO: 8-Methylthiooctyl-GLS
BGLU: Beta Glucosidase
ED: Extended darkness
GLS: Glucosinolates
I3M: Indol-3-ylmethyl-GLS
ITC: Isothiocyanate
LD: Long
MVP1: Modified vacuolar phenotype 1
SD: Short day
TGG: Thioglucoside glucohydrolase

## References

[1] D’Auria JC, Gershenzon J. The secondary metabolism of Arabidopsis thaliana: Growing like a weed. Curr. Opin. Plant Biol. 2005;8(3 SPEC. ISS.):308–316. 10.1016/j.pbi.2005.03.012 15860428

[2] Brown PD, Tokuhisa JG, Reichelt M, Gershenzon J. Variation of Glucosinolate Accumulation among Different Organs and Developmental Stages of Arabidopsis thaliana. Phytochemistry. 2003;62(MARCH):471–481. 10.1016/S0031-9422(02)00549-6 12620360

[3] Bones AM, Rossiter JT. The myrosinase-glucosinolate system, its organisation and biochemistry. Physiol. Plant. 1996;97(1):194–208. 10.1111/j.1399-3054.1996.tb00497.x

[4] Kliebenstein DJ, Kroymann J, Brown P, Figuth A, Pedersen D, Gershenzon J, et al Genetic control of natural variation in Arabidopsis glucosinolate accumulation. Plant Physiol. 2001;126(June):811–825. 10.1104/pp.126.2.811 11402209 PMC111171

[5] Brown PD, Morra MJ. Control of Soil-Borne Plant Pests Using Glucosinolate-Containing Plants. Adv. Agron. 1997;61(C):167–231. 10.1016/S0065-2113(08)60664-1

[6] Fahey JW, Zalcmann AT, Talalay P. The chemical diversity and distribution of glucosinolates and isothiocyanates amoung plants. Phytochemistry. 2001;56:5–51. 10.1016/S0031-9422(00)00316-2 11198818

[7] Das S, Tyagi AK, Kaur H. Cancer modulation by glucosinolates: A review. Curr. Sci. 2000;79(12):1665–1671.

[8] Hayes JD, Kelleher MO, Eggleston IM. The cancer chemopreventive actions of phytochemicals derived from glucosinolates. Eur. J. Nutr. 2008;47(SUPPL. 2):73–88. 10.1007/s00394-008-2009-8 18458837

[9] Hecht SS. Chemoprevention of Cancer by Isothiocyanates, Modifiers of Carcinogen Metabolism. J. Nutr. 1999;129:768–774. 10.1093/jn/129.3.768S 10082787

[10] Halkier BA, Gershenzon J. Biology and Biochemistry of Glucosinolates. Annu. Rev. Plant Biol. 2006;57(1):303–333. 10.1146/annurev.arplant.57.032905.105228 16669764

[11] Rask L, Andréasson E, Ekbom B, Eriksson S, Pontoppidan B, Meijer J. Myrosinase: Gene family evolution and herbivore defense in Brassicaceae. Plant Mol. Biol. 2000;42(1):93–113. 10.1023/A:1006380021658 10688132

[12] Wittstock U, Burow M. Glucosinolate Breakdown in Arabidopsis: Mechanism, Regulation and Biological Significance. Arab. B. 2010; 10.1199/tab.0134 22303260 PMC3244901

[13] Koroleva OA, Davies A, Deeken R, Thorpe MR, Tomos AD, Hedrich R. Identification of a new glucosinolate-rich cell type in Arabidopsis flower stalk. Plant Physiol. 2000;124(2):599–608. 10.1104/pp.124.2.599 11027710 PMC59166

[14] Ueda H, Nishiyama C, Shimada T, Koumoto Y, Hayashi Y, Kondo M, et al AtVAM3 is required for normal specification of idioblasts, myrosin cells. Plant Cell Physiol. 2006;47(1):164–175. 10.1093/pcp/pci232 16306062

[15] Burow M, Halkier BA. How does a plant orchestrate defense in time and space? Using glucosinolates in Arabidopsis as case study. Curr. Opin. Plant Biol. 2017;38:142–147. 10.1016/j.pbi.2017.04.009 28575680

[16] Grubb CD, Abel S. Glucosinolate metabolism and its control. Trends Plant Sci. 2006;11(2):89–100. 10.1016/j.tplants.2005.12.006 16406306

[17] Neilson EH, Goodger JQD, Woodrow IE, Møller BL. Plant chemical defense: At what cost? Trends Plant Sci. 2013;18(5):250–258. 10.1016/j.tplants.2013.01.001 23415056

[18] Yan X, Chen S. Regulation of plant glucosinolate metabolism. Planta. 2007;226(6):1343–1352. 10.1007/s00425-007-0627-7 17899172

[19] Rosa EAS. Daily variation in glucosinolate concentrations in the leaves and roots of cabbage seedlings in two constant temperature regimes. J. Sci. Food Agric. 1997;73(3):364–368. 10.1002/(SICI)1097-0010(199703)73:3<364::AID-JSFA742>3.0.CO;2-O

[20] Rosa EAS, Heaney RK, Rego FC, Fenwick GR. The variation of glucosinolate concentration during a single day in young plants of Brassica oleracea var Acephala and Capitata. J. Sci. Food Agric. 1994;66(4):457–463. 10.1002/jsfa.2740660406

[21] Petersen BL, Chen S, Hansen CH, Olsen CE, Halkier BA. Composition and content of glucosinolates in developing Arabidopsis thaliana. Planta. 2002;214(4):562–571. 10.1007/s004250100659 11925040

[22] Barth C, Jander G. Arabidopsis myrosinases TGG1 and TGG2 have redundant function in glucosinolate breakdown and insect defense. Plant J. 2006;46(4):549–562. 10.1111/j.1365-313X.2006.02716.x 16640593

[23] Huseby S, Koprivova A, Lee BR, Saha S, Mithen R, Wold AB, et al Diurnal and light regulation of sulphur assimilation and glucosinolate biosynthesis in Arabidopsis. J. Exp. Bot. 2013;64(4):1039–1048. 10.1093/jxb/ers378 23314821 PMC3580815

[24] Goodspeed D, Liu JD, Chehab EW, Sheng ZJ, Francisco M, Kliebenstein DJ, Braam J. (2013). Postharvest Circadian Entrainment Enhances Crop Pest Resistance and Phytochemical Cycling. Current Biology 2013;23, 1235–1241. 10.1016/j.cub.2013.05.034 23791724

[25] Falk KL, Tokuhisa JG, Gershenzon J. The effect of sulfur nutrition on plant glucosinolate content: Physiology and molecular mechanisms. Plant Biol. 2007;9(5):573–581. 10.1055/s-2007-965431 17853357

[26] Hirai MY, Yano M, Goodenowe DB, Kanaya S, Kimura T, Awazuhara M, et al Integration of transcriptomics and metabolomics for understanding of global responses to nutritional stresses in Arabidopsis thaliana. Proc. Natl. Acad. Sci. 2004;101(27):10205–10210. 10.1073/pnas.0403218101 15199185 PMC454188

[27] Kutz A, Müller A, Hennig P, Kaiser WM, Piotrowski M, Weiler EW. A role for nitrilase 3 in the regulation of root morphology in sulphur-starving Arabidopsis thaliana. Plant J. 2002;30(1):95–106. 10.1046/j.1365-313X.2002.01271.x 11967096

[28] Zhao F, Evans EJ, Bilsborrow PE, Syers JK. Influence of nitrogen and sulphur on the glucosinolate profile of rapeseed (Brassica napus). J. Sci. Food Agric. 1994;64(3):295–304. 10.1002/jsfa.2740640309

[29] Nakano RT, Yamada K, Bednarek P, Nishimura M, Hara-Nishimura I. ER bodies in plants of the Brassicales order: biogenesis and association with innate immunity. Front. Plant Sci. 2014;5(March):1–17. 10.3389/fpls.2014.00073 24653729 PMC3947992

[30] Nakano RT, Piślewska-Bednarek M, Yamada K, Edger PP, Miyahara M, Kondo M, et al PYK10 myrosinase reveals a functional coordination between endoplasmic reticulum bodies and glucosinolates in Arabidopsis thaliana. Plant J. 2017;89(2):204–220. 10.1111/tpj.13377 27612205

[31] Ogasawara K, Yamada K, Christeller JT, Kondo M, Hatsugai N, Hara-Nishimura I, et al Constitutive and Inducible ER bodies of Arabidopsis thaliana Accumulate Distinct β-Glucosidases. Plant Cell Physiol. 2009;50(3):480–488. 10.1093/pcp/pcp007 19147648

[32] Yamada K, Nagano AJ, Ogasawara K, Hara-Nishimura I, Nishimura M. The ER body, a new organelle in Arabidopsis thaliana, requires NAI2 for its formation and accumulates specific β-glucosidases. Plant Signal. Behav. 2009;4(9):849–852. 10.4161/psb.4.9.9377 19847124 PMC2802796

[33] Lambrix V, Reichelt M, Mitchell-Olds T, Kliebenstein DJ, Gershenzon J. The Arabidopsis epithiospecifier protein promotes the hydrolysis of glucosinolates to nitriles and influences Trichoplusia ni herbivory. Plant Cell. 2001; 13: 2793–2807. 10.1105/tpc.010261 11752388 PMC139489

[34] Burow M, Rice M, Hause B, Gershenzon J, Wittstock U. Cell- and tissue-specific localization and regulation of the epithiospecifier protein in Arabidopsis thaliana. Plant Mol Biol. 2007; 64: 173–185. 10.1007/s11103-007-9143-1 17390109

[35] Wentzell AM, Kliebenstein DJ. Genotype, age, tissue, and environment regulate the structural outcome of glucosinolate activation. Plant Physiol. 2008; 147: 415–428. 10.1104/pp.107.115279 18359845 PMC2330308

[36] Burow M, Wittstock U. Regulation and function of specifier proteins in plants. Phytochem Rev. 2009; 8: 87–99. 10.1007/s11101-008-9113-5

[37] Burow M, Losansky A, Müller R, Plock A, Kliebenstein DJ, Wittstock U. The genetic basis of constitutive and herbivore-induced ESP-independent nitrile formation in Arabidopsis. Plant Physiol. 2009; 149: 561–574. 10.1104/pp.108.130732 18987211 PMC2613743

[38] James DC, Rossiter JT. Development and characteristics of myrosinase in Brassica napus during early seedling growth. Physiol. Plant. 1991;82:163–170.

[39] Schonhof I, Blankenburg D, Müller S, Krumbein A. Sulfur and nitrogen supply influence growth, product appearance, and glucosinolate concentration of broccoli. J. Plant Nutr. Soil Sci. 2007;170(1):65–72. 10.1002/jpln.200620639

[40] Araújo WL, Tohge T, Ishizaki K, Leaver CJ, Fernie AR. Protein degradation—an alternative respiratory substrate for stressed plants. Trends Plant Sci. 2011;16(9):489–498. 10.1016/j.tplants.2011.05.008 21684795

[41] Hildebrandt TM, Nunes Nesi A, Araújo WL, Braun HP. Amino Acid Catabolism in Plants. Mol. Plant. 2015;8(11):1563–1579. 10.1016/j.molp.2015.09.005 26384576

[42] Tohge T, Fernie AR. Combining genetic diversity, informatics and metabolomics to facilitate annotation of plant gene function. Nat. Protoc. 2010;5(6):1210–1227. 10.1038/nprot.2010.82 20539294

[43] Tohge T, Wendenburg R, Ishihara H, Nakabayashi R, Watanabe M, Sulpice R, et al Characterization of a recently evolved flavonol-phenylacyltransferase gene provides signatures of natural light selection in Brassicaceae. Nat. Commun. 2016;7:12399 10.1038/ncomms12399 27545969 PMC4996938

[44] Nakabayashi R, Kusano M, Kobayashi M, Tohge T, Yonekura-Sakakibara K, Kogure N, et al Metabolomics-oriented isolation and structure elucidation of 37 compounds including two anthocyanins from Arabidopsis thaliana. Phytochemistry 2009;70(8):1017–1029. 10.1016/j.phytochem.2009.03.021 19497599

[45] Palmieri S, Leoni O, Iori R. A steady-state kinetics study of myrosinase with direct ultraviolet spectrophotometric assay. Anal Biochem. 1982; 123: 320–324. 10.1016/0003-2697(82)90452-3 7125206

[46] Van Slyke DD, Hiller A. Determination of ammonia in blood. J. Biol. Chem. 1933;102:499–504.

[47] Boyes DC, Zayed AM, Ascenzi R, McCaskill AJ, Hoffman NE, Davis KR, et al Growth Stage-Based Phenotypic Analysis of Arabidopsis: A Model for High Throughput Functional Genomics in Plants. Plant Cell 2001;13(7):1499–1510. 11449047 10.1105/TPC.010011 PMC139543

[48] Izumi M, Hidema J, Makino A, Ishida H. Autophagy Contributes to Nighttime Energy Availability for Growth in Arabidopsis. Plant Physiol. 2013;161(4):1682–1693. 10.1104/pp.113.215632 23457226 PMC3613448

[49] Peng C, Uygun S, Shiu S-H, Last Robert L. The Impact of the Branched-Chain Ketoacid Dehydrogenase Complex on Amino Acid Homeostasis in Arabidopsis. Plant Physiol. 2015; 169: 1807–1820. 10.1104/pp.15.00461 25986129 PMC4634046

[50] Liu JD, Goodspeed D, Sheng ZJ, Li BH, Yang YR, Kliebenstein DJ, Braam J. Keeping the rhythm: light/dark cycles during postharvest storage preserve the tissue integrity and nutritional content of leafy plants. Bmc Plant Biology 2015; 15 10.1186/s12870-015-0474-9 25879637 PMC4396971

[51] Janowitz T, Trompetter I, Piotrowski M. Evolution of nitrilases in glucosinolate-containing plants. Phytochemistry. 2009; 70: 1680–1686. 10.1016/j.phytochem.2009.07.028 19698961

[52] Howden AJM, Preston GM. Nitrilase enzymes and their role in plant-microbe interactions. Microb Biotechnol. 2009; 2: 441–451. 10.1111/j.1751-7915.2009.00111.x 21255276 PMC3815905

[53] Matile P. The mustard oil bomb compartmentation of the myrosinase system. Biochem. und Physiol. der Pflanz. 1980;175(8–9):722–731.

[54] Koroleva OA, Cramer R. Single-cell proteomic analysis of glucosinolate-rich S-cells in Arabidopsis thaliana. Methods 2011;54(4):413–423. 10.1016/j.ymeth.2011.06.005 21708264

[55] Nour-Eldin HH, Andersen TG, Burow M, Madsen SR, Jørgensen ME, Olsen CE, et al NRT/PTR transporters are essential for translocation of glucosinolate defence compounds to seeds. Nature. 2012; 488: 531–534. 10.1038/nature11285 22864417

[56] Andersen TG, Nour-Eldin HH, Fuller VL, Olsen CE, Burow M, Halkier BA. Integration of biosynthesis and long-distance transport establish organ-specific glucosinolate profiles in vegetative Arabidopsis. Plant Cell. 2013; 25: 3133–3145. 10.1105/tpc.113.110890 23995084 PMC3784604

[57] Hansen BG, Kliebenstein DJ, Halkier BA. Identification of a flavin-monooxygenase as the S-oxygenating enzyme in aliphatic glucosinolate biosynthesis in Arabidopsis. Plant J. 2007; 50: 902–910. 10.1111/j.1365-313X.2007.03101.x 17461789

[58] Li J, Hansen BG, Ober JA, Kliebenstein DJ, Halkier BA. Subclade of flavin-monooxygenases involved in aliphatic glucosinolate biosynthesis. Plant Physiol. 2008; 148: 1721–1733. 10.1104/pp.108.125757 18799661 PMC2577257

[59] Kong W, Li J, Yu Q, Cang W, Xu R, Wang Y, et al Two Novel Flavin-Containing Monooxygenases Involved in Biosynthesis of Aliphatic Glucosinolates. Front Plant Sci. 2016; 7: 1292 10.3389/fpls.2016.01292 27621741 PMC5003058

[60] Chung WC, Huang HC, Chiang BT, Huang HC, Huang JW. Inhibition of soil-borne plant pathogens by the treatment of sinigrin and myrosinases released from reconstructed Escherichia coli and Pichia pastoris. Biocontrol Sci. Technol. 2005;15(5):455–465. 10.1080/09583150500086607

[61] Agerbirk N, De Vos M, Kim JH, Jander G. Indole glucosinolate breakdown and its biological effects. Phytochem. Rev. 2009;8(1):101–120. 10.1007/s11101-008-9098-0

[62] Ljung K, Hull AK, Kowalczyk M, Marchant A, Celenza J, Cohen JD, et al Biosynthesis, conjugation, catabolism and homeostasis of indole-3-acetic acid in Arabidopsis thaliana. Plant Mol. Biol. 2002;50(2):309–332. 10.1023/A:1016024017872 12175022

[63] Woodward AW, Bartel B. Auxin: Regulation, action, and interaction. Ann. Bot. 2005;95(5):707–735. 10.1093/aob/mci083 15749753 PMC4246732

[64] Cheung KL, Kong A-N. Molecular Targets of Dietary Phenethyl Isothiocyanate and Sulforaphane for Cancer Chemoprevention. AAPS J. 2010;12(1):87–97. 10.1208/s12248-009-9162-8 20013083 PMC2811646

