# Supplementary figures and images for: Extended darkness induces internal turnover of glucosinolates in Arabidopsis thaliana leaves
Source: PLoS One. 2018 Aug 9;13(8):e0202153. doi: 10.1371/journal.pone.0202153 (PMC6084957; doi:10.1371/journal.pone.0202153)

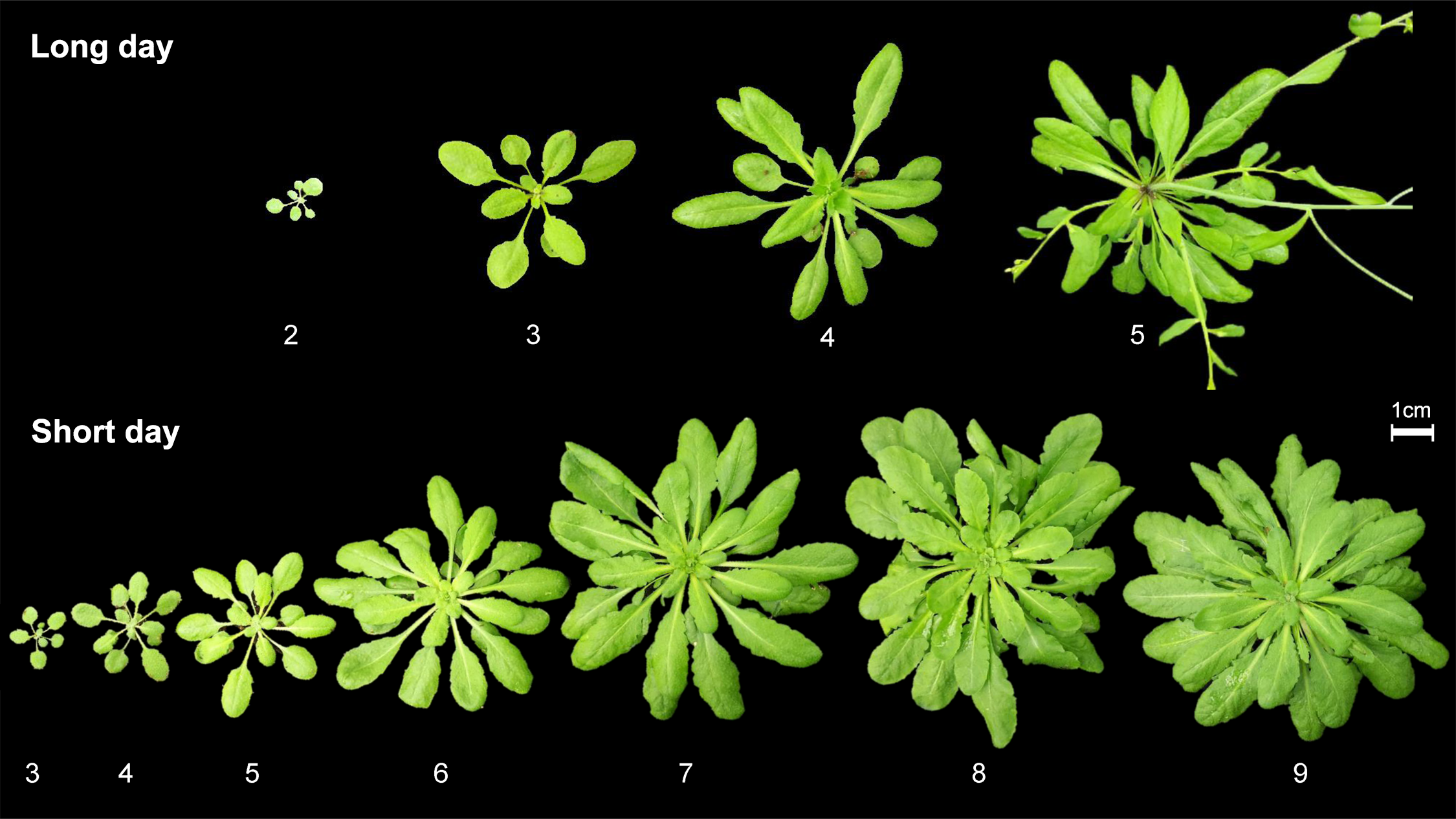

Supplement: S1 Fig — The plants were grown at 22 °C, 85 μmol s−1 m−2 light and 65% humidity for 16 h light/8 h dark (long day) and 8 h light/16 h dark (short day). Numbers indicate the age of the plants in weeks. (TIF) [file pone.0202153.s001.tif]

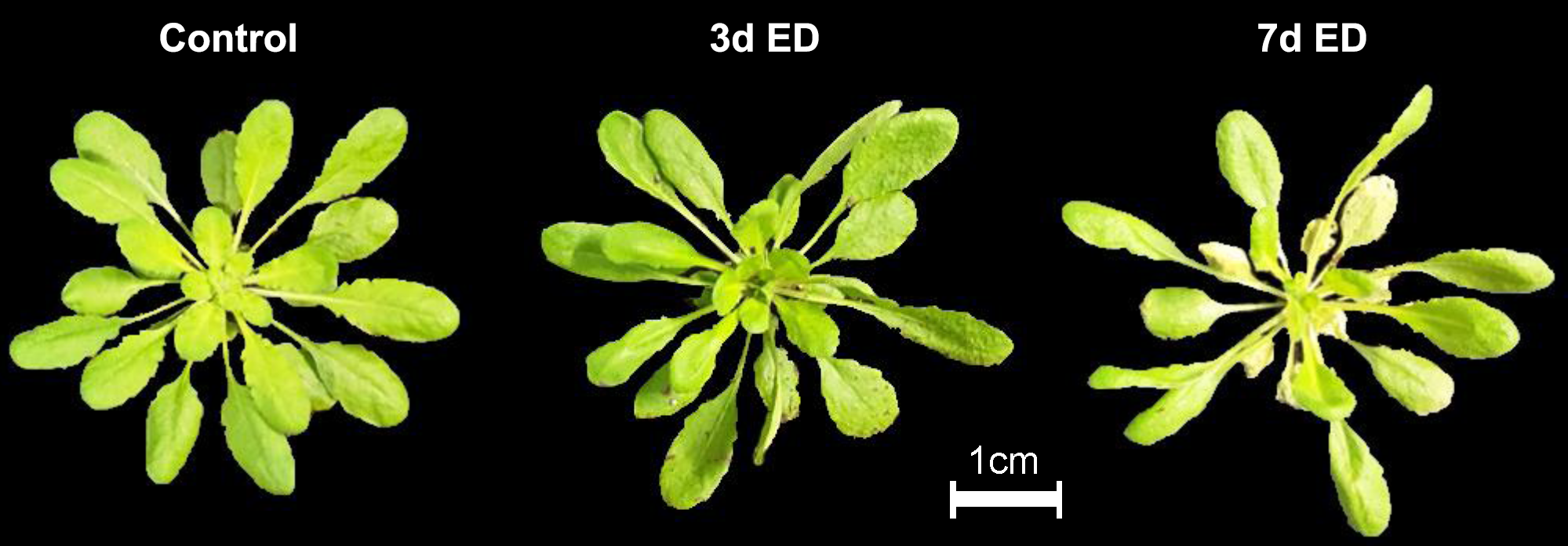

Supplement: S2 Fig — Phenotype of six week old plants grown under short-day conditions (22 °C, 85 μmol s−1 m−2 light, 65% humidity) and transferred to darkness (22 °C, 65% humidity) for 3 d and 7 d. (TIF) [file pone.0202153.s002.tif]

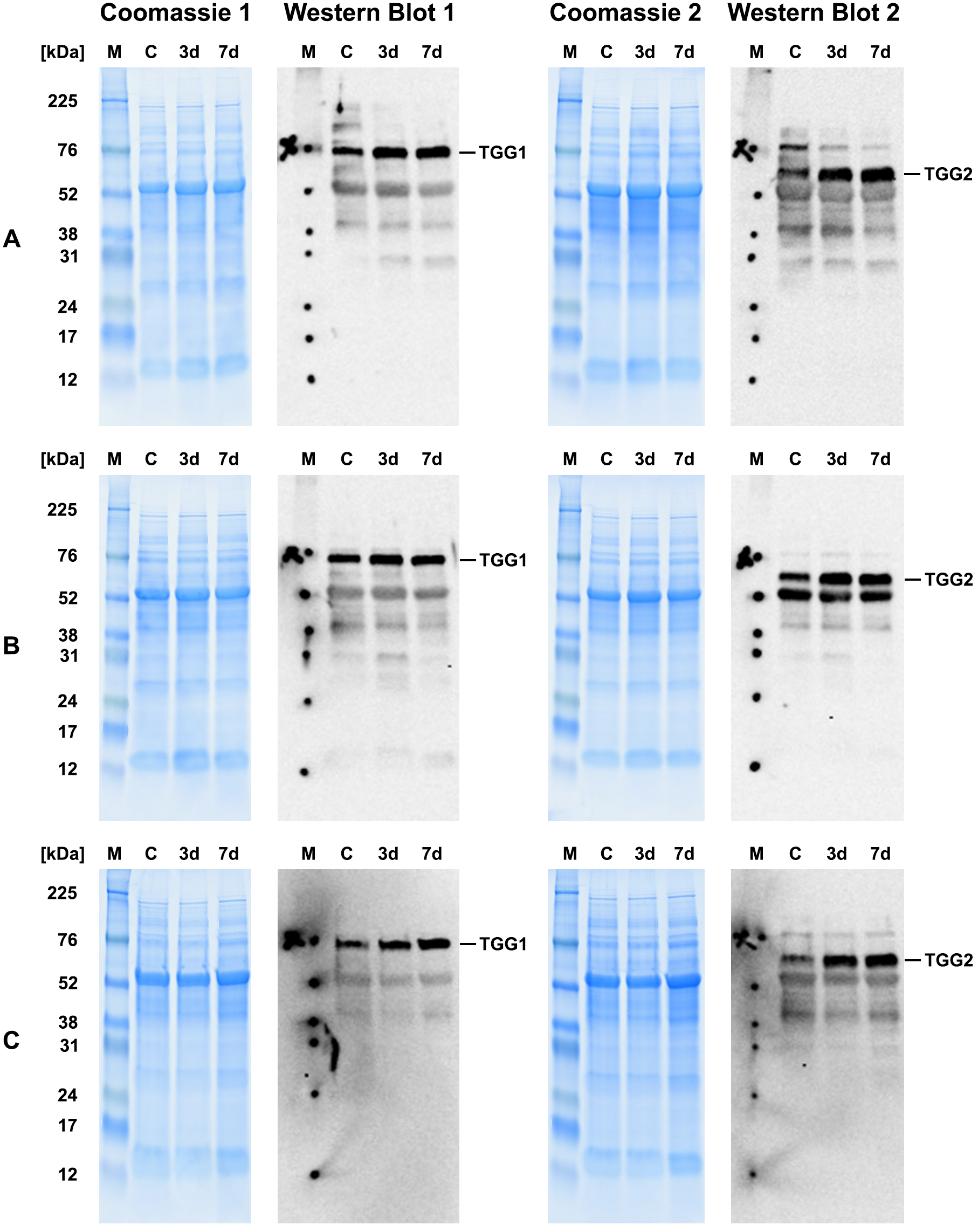

Supplement: S3 Fig — Leaves of six-week-old plants, grown under short day conditions, were harvested after 0 d (Control), 3 d (3 d ED) and 7 d (7 d ED) of extended darkness, directly frozen and ground to powder. 6 μg of denatured leaf extract was load on a SDS-PAGE and further either stained with Coomassie brilliant blue (Coomassie 1/2) or transferred to a nitrocellulose membrane (Western Blot 1/2). Specific antibodies against the myrosinase isoforms TGG1 and TGG2 were used. M: Amersham ECL High-Range Rainbow marker (GE Healthcare). A-C: three biological replicates. The samples for Coomassie-stain and the Western Blots were prepared on one gel for TGG1 and TGG2, respectively. (TIF) [file pone.0202153.s003.tif]

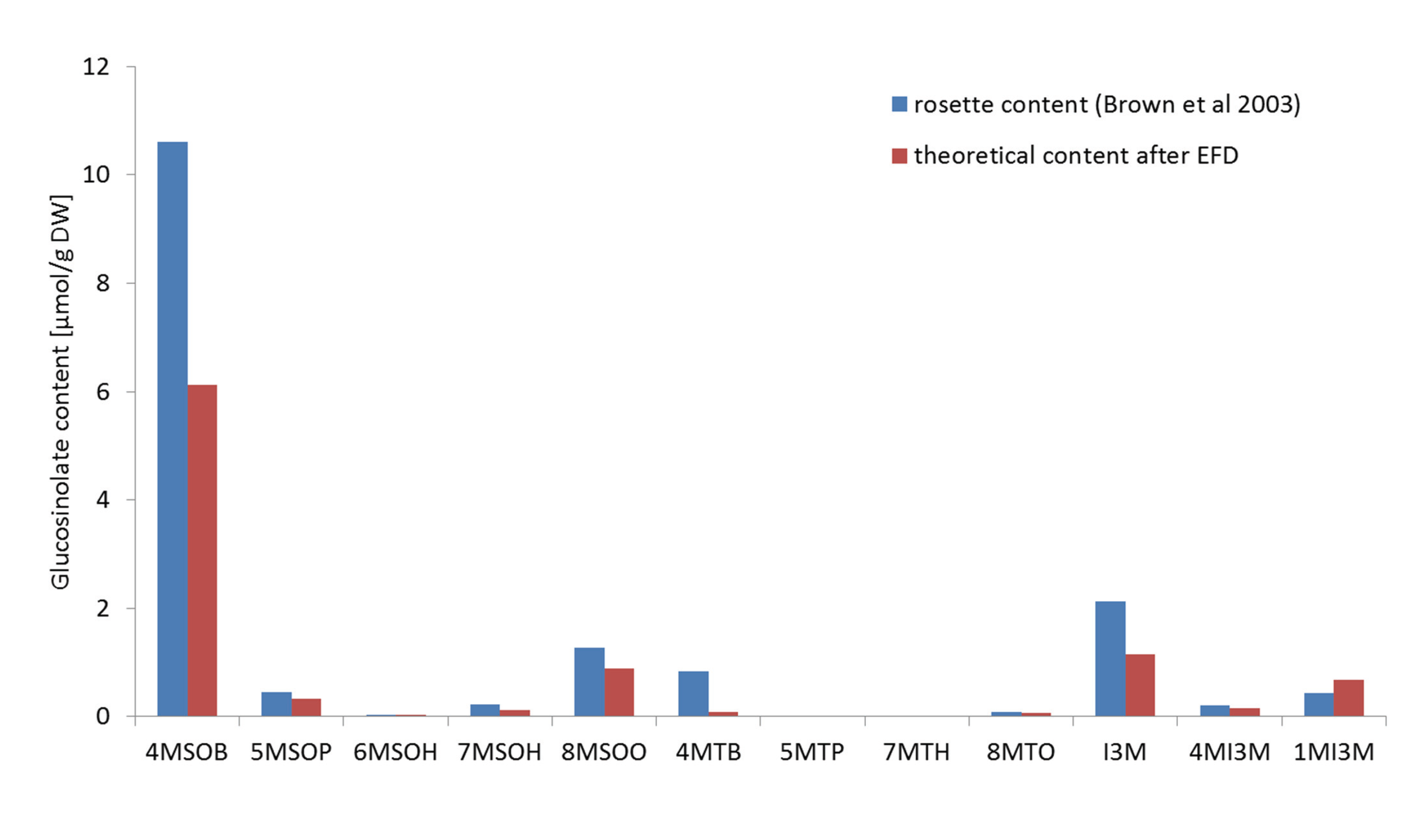

Supplement: S4 Fig — The GLS profile of rosette leaves in the vegetative state published by Brown et al. 2003 [2] (blue bars) was used to calculate the theoretical content of each GLC analyzed in this study after 7d of extended darkness (red bars). Relative changes in GLS levels including standard deviations and statistics are shown in Fig 5. 4MSOB (4-Methylsulfinylbutyl-GLS); 5MSOP (5-Methylsulfinylpentyl-GLS); 6MSOH (6-Methylsulfinylhexyl-GLS); 7MSOH (7-Methylsulfinylheptyl); 8MSOO (8-Methylsulfinyloctyl); 4MTB (4-Methylthiobutyl-GLS); 5MTP (5-Methylthiopentyl-GLS); 7MTH (7-Methylthioheptyl-GLS); 8MTO (8-Methylthiooctyl-GLS); I3M (Indol-3-ylmethyl-GLS); 4MI3M (4-Methoxy-indol-3-ylmethyl-GLS); 1MI3M (N-Methoxy-indol-3-ylmethyl-GLS). (TIF) [file pone.0202153.s004.tif]
